# Precision controlled atomic resolution scanning transmission electron microscopy using spiral scan pathways

**DOI:** 10.1038/srep43585

**Published:** 2017-03-08

**Authors:** Xiahan Sang, Andrew R. Lupini, Jilai Ding, Sergei V. Kalinin, Stephen Jesse, Raymond R. Unocic

**Affiliations:** 1 Center for Nanophase Materials Sciences Oak Ridge National Laboratory, Oak Ridge, TN, 37831, USA; 2 Institute for Functional Imaging of Materials Oak Ridge National Laboratory, Oak Ridge, TN, 37831, USA; 3 Materials Science and Technology Division Oak Ridge National Laboratory, Oak Ridge, TN, 37831, USA

## Abstract

Atomic-resolution imaging in an aberration-corrected scanning transmission electron microscope (STEM) can enable direct correlation between atomic structure and materials functionality. The fast and precise control of the STEM probe is, however, challenging because the true beam location deviates from the assigned location depending on the properties of the deflectors. To reduce these deviations, *i*.*e*. image distortions, we use spiral scanning paths, allowing precise control of a sub-Å sized electron probe within an aberration-corrected STEM. Although spiral scanning avoids the sudden changes in the beam location (fly-back distortion) present in conventional raster scans, it is not distortion-free. “Archimedean” spirals, with a constant angular frequency within each scan, are used to determine the characteristic response at different frequencies. We then show that such characteristic functions can be used to correct image distortions present in more complicated constant linear velocity spirals, where the frequency varies within each scan. Through the combined application of constant linear velocity scanning and beam path corrections, spiral scan images are shown to exhibit less scan distortion than conventional raster scan images. The methodology presented here will be useful for *in situ* STEM imaging at higher temporal resolution and for imaging beam sensitive materials.

Scanning transmission electron microscopy (STEM) is an extremely versatile tool for materials characterization, offering sub-Å spatial resolution and sub-second temporal resolution^1^,^2^,^3^. The electron beam formed in an C_s_-aberration-corrected STEM is the smallest available probe that can be accessed and controlled for scientific research^2^,^4^,^5^. Spectroscopy data such as energy dispersive X-ray (EDS) and electron energy loss spectroscopy (EELS) can be simultaneously acquired using the same local probe^6^,^7^,^8^,^9^. To date, the only scan path that has been widely adopted in imaging mode is the raster scan, whereby the electron beam scans from left to right rapidly and top to bottom more slowly. The raster scan has a primary advantage in that the signal acquired at each scan position can readily be assigned to a pixel, which is the basic unit in image storage and analysis techniques. However, distortions can arise when the true probe position does not match the desired position, for example because of drift, instabilities, or fly-back distortion resulting from the sudden change of beam location from the end of a line to the beginning of the next line^10^,^11^. Therefore, post-acquisition scan distortion correction methods have been proposed based on analyzing features of the STEM images^11^,^12^,^13^,^14^,^15^,^16^,^17^. For example, by changing the scan direction for a series of images, the revolving STEM (RevSTEM) method can be used to efficiently measure drift vectors and correct drift distortions, enabling picometer-level measurements of the lattice constants^18^ and local displacements^14^. Similarly, non-linear drift distortion can be corrected from pairs of orthogonal scanned STEM images^17^. The fly-back distortions, however, are difficult to correct especially for fast raster scans.

Clearly, greater flexibly and control of the electron beam with picometer-level precision is required to achieve atomic-scale imaging and spectroscopy at sub-second temporal resolution. Alternatives to conventional raster scans have previously been implemented in scanning tunneling microscopy (STM) and atom force microscopy (AFM), where a probe is physically moved across the samples surface in non-raster scan paths, in order to correct for sample drift^19^ or to enable fast image acquisition^20^. Recently, a general-scan STEM (G-STEM) technique has been proposed to scan the beam along spiral pathways, while still maintaining atomic resolution^15^. Spiral scan paths are smooth and differentiable to arbitrary order; as such, we can avoid the scan time wasted on fly-back that is used to reduce distortion from the sudden beam position changes in raster scans, thereby potentially improving temporal resolution and reducing beam irradiation. Details can be found in the previous paper^15^ and in Fig. 1. However, inherent distortions arise from spiral scans and these distortions are directly related to the error in the beam position. Understanding the scan distortion for simple spirals thus may lead to better control of the electron beam for more complicated scan pathways.

Here we demonstrate, for Archimedean spirals with constant scan frequencies, how the scan distortion can be modeled using two-dimensional (2D) affine transformations that are solely determined by the scan frequency. We then show its general applicability in other spiral scan modes with demonstration on constant linear velocity (CLV) spiral scans. This beam control method, combined with optimized spiral scan paths, yields atomic-resolution STEM images free of major scan distortions when images are acquired within a short frame time of 0.05 s, while raster scan images acquired using the same conditions exhibit significant fly-back distortions across the whole image frame.

## Results and Discussion

### Actual beam position and nominal beam position

Due to limitations in the scan system, the actual *i*^th^ beam position, *L*_*i*_ = (*x*_*i*_, *y*_*i*_), deviates from the nominal *i*^th^ beam position, 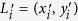. Excluding random noise and drift, the systemic difference, Δ*L*_*i*_, between *L*_*i*_ and 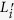 is related to the details of the scan system and the rate of beam motion (magnetic scan coils suffer from inductance hysteresis, scan plates have capacitance, and amplifiers have a finite time-response). For raster scans, excluding the transition period when the beam flies from the end of the line to the start of the next line, the beam essentially moves at a constant velocity. Therefore, the distortion (Δ*L*_*i*_ = *L*_*i*_ − 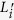) might be expected to be the same for all the pixels, as the beam moves at the same speed from left to right, resulting in a uniform and rigid translation between the STEM image and the actual sample area.

**Figure 2:**
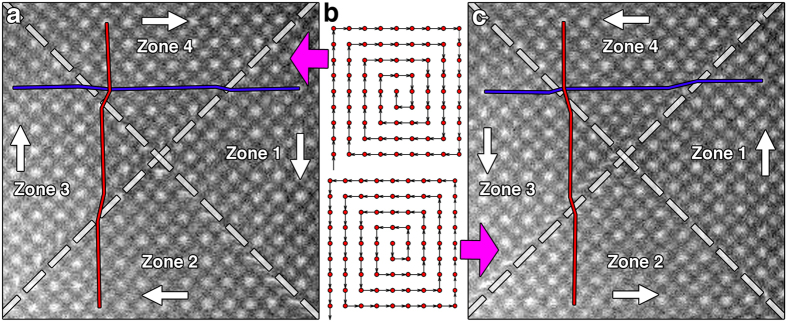
Experimental single-frame STEM images acquired along [001] SrTiO_3_ using square spiral scan paths where the beam moves clockwise (**a**) and counter-clockwise (**c**). (**b**) Schematics of the clockwise scan path (upper) and the counter-clockwise scan path (lower). The beam scan directions are indicated by the white arrows. Zones with uniform velocity are separated by the dashed lines. The red and blue lines indicate atom columns that should belong to the same lattice plane.

This translation can be visualized by using a square spiral scan path, by moving the probe alternatively along horizontal and vertical directions to fill the area in a spiral fashion with a constant speed (Fig. 2b). Figure 2a and c show the reconstructed images acquired with a frame time of 2 s for a clockwise scan direction and a counter-clockwise scan direction, respectively, from [001] SrTiO_3_ (STO). STO has a perovskite crystal structure and in STEM images the bright and weak spots correspond to Sr and Ti atom columns, respectively (the weak O atom columns are not visible). The scan directions are indicated by the white arrows in Fig. 2a and c and both STEM images are divided into four quadrants, labeled as zones 1–4. Within each quadrant, the beam moves at a constant velocity and the corresponding image area does not exhibit any unexpected distortion. However, at the boundaries between the zones, significant distortions arise due to the sharp 90° change in the beam direction, which manifests as a constant displacement between two zones in Fig. 2 for the relatively slow scan, and more obvious distorted bands (see Supplementary Fig. S1) for faster scans. The displacements are indicated by the kinks in the red and blue lines that are drawn across the boundary to connect atom columns from the same lattice plane in the STEM images in Fig. 2. The observed boundary displacements can be directly correlated with the scan direction. For example, zone 4 in Fig. 2a moves to the right relative to zone 3, and so on.

The translational distortion is universal for the raster scan^21^, i.e., the STEM image is misaligned with the nominal sample area by a translational vector, the magnitude of which depends on the beam dwell time and the scan frequency (see Supplementary Fig. S1). This scan distortion must be considered when selecting areas for high-resolution spectroscopic analysis, such as for a single atomic column selected from an as-acquired or frozen STEM image displayed on the screen. The displacement of the image along the scan direction indicates that the actual beam location, *L*_*i*_, lags behind the assigned beam location, 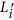. This lag is expected to depend on the beam acceleration and must be accounted for in order to establish more precise control of the true probe location. Archimedean spiral scans with different preset angular velocities (frequencies) will be used to quantitatively measure the distortion.

### Transformation Matrices Determined from Archimedean spirals

The general form of spiral curves can be written as:


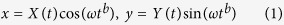


when *X*(*t*) = *Y*(*t*) = *t* and *b* = 1, we have the Archimedean spiral with a constant angular velocity, *ω*. The Archimedean spiral, as its name suggests, has been known for thousands of years and the details of its properties are discussed elsewhere^15^,^22^. For the STEM scan coils, a constant angular velocity means the control signal has a constant frequency, *f* = *ω*/2*π*^15^. Positive frequency is defined as when the beam rotates counter-clockwise and negative frequency is when the beam rotates clockwise. As the beam spirals outward away from the center, the scan step size increases linearly as a function of 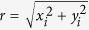. Therefore, the beam moves much faster near the edges of the scan area than in the center. The sampling density is thus much higher in the center of the scan than on the edge, resulting in lower SNR on the edge and a higher beam dose in the center, the latter of which can increase sample damage.

**Figure 3:**
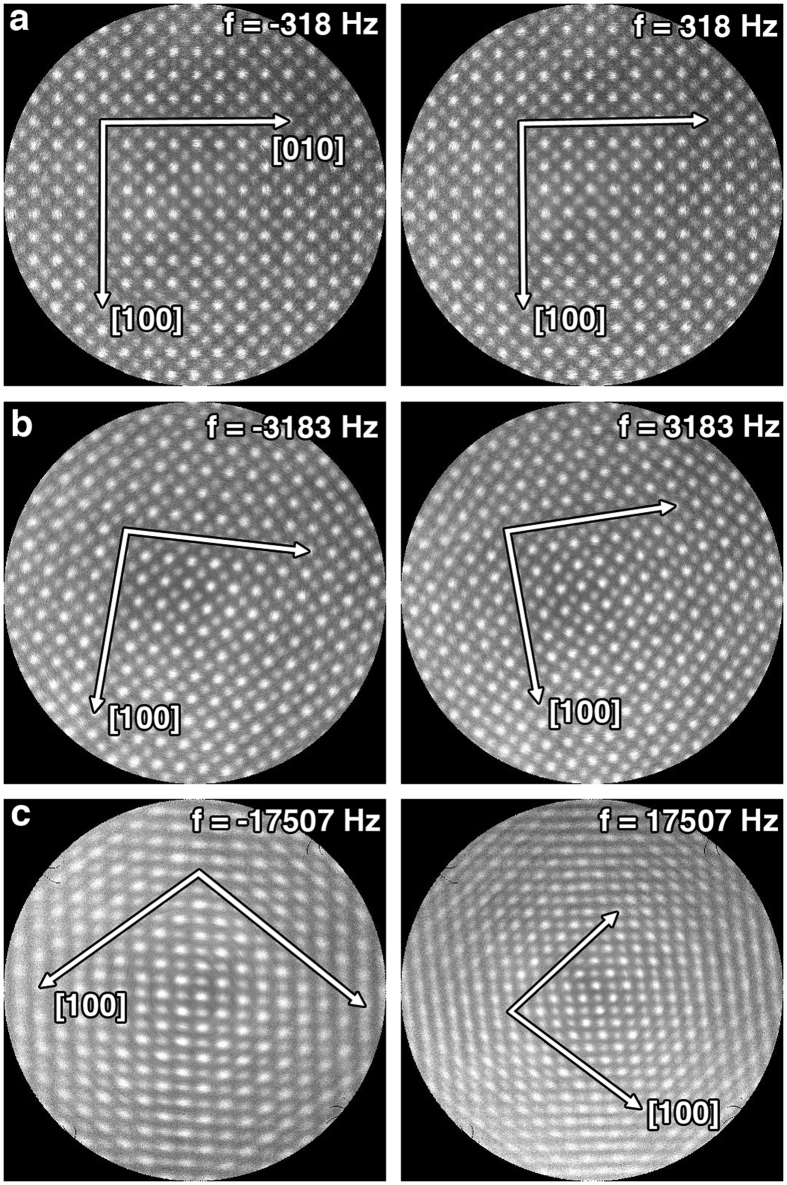
Reconstructed G-STEM images acquired from [001] oriented STO using Archimedean spiral scans at different frequencies (**a**) *f* = ±318 Hz, (**b**) *f* = ±3,183 Hz, and (**c**) *f* = ±17,507 Hz. The two cube axes, [100] and [010] directions, are indicated by white arrows. The ‘[010]’ label is shown for *f* = −318 Hz.

Archimedean spiral scans with *f* varying from 0.3 kHz to 76 kHz were used to acquire G-STEM images of an STO sample aligned along the [001] zone axis. The scan area of the Nion SuperScan system was set to 8 nm × 8 nm and the two cube axes were aligned with the horizontal and vertical directions using the scan rotation control. Each dataset was acquired using a 1 s frame time at a 2 MHz readout frequency. The voltage range applied from the external scan system was [−2, 2] V for both x and y directions. The G-STEM images were reconstructed on a 512 × 512 grid to form a 512 × 512 pixel image. Figure 3 shows [001] STO STEM images reconstructed from Archimedean spiral scans for (a) *f* = ±318 Hz, (b) *f* = ±3,183 Hz, and (c) *f* = ±17507 Hz. The scan rotation was aligned with the [010] lattice vector along the fast raster-scan direction and the [100] lattice vector along the slow raster-scan direction. The distortion for each scan condition is observed from the scan rotation, change of angle, or change in length of the two lattice vectors, [100] and [010] (indicated by white arrows in Fig. 3). Although the six experimental images shown in Fig. 3 exhibit different distortions, the distortion within each image is approximately a 2D affine transformation, as the two basic lattice vectors remain straight in all six images. However, the lattice vector length and the angle between [100] and [010] change as *f* changes. This is illustrated for the case of the spiral scan with *f* = ±318 Hz, where the two experimental STEM images shown in Fig. 3a appear similar but show a slight difference in relative rotation. The rotation is more evident for *f* = ±3,183 Hz (Fig. 3b). The image rotation direction also coincides with the beam rotation direction, which is similar to the image distortion observed for the square spiral case. A shearing distortion is also observed where the angle between [100] and [010] is less than 90° for positive *f* and larger than 90° for negative *f*. The shearing distortion is most pronounced for *f* = ±17,507 Hz, as shown in Fig. 3c. The different behavior for negative and positive *f*, and the observed shearing distortion, indicate the possible coupling between scan coil *x* and scan coil *y*.

Figure 3 suggests that the key problem for accurate beam control is to determine the frequency-dependent image distortion that is described by the affine transformation matrix, *T*^*f*^. Two non-collinear axes 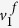 and 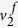 for each *f* are used as the base vectors, thus, *T*^*f*^ is defined as,


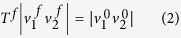


Here 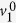 and 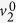 are the two base vectors when *f* = 0. However, the *f* = 0 case cannot be directly measured, because by definition the Archimedean spiral must have a non-zero *ω*. One solution is to assume the transformation matrices for small *f* are inverse matrices; for example,


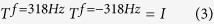


where *I* is the identity matrix. This approximation works well because for small *f*, the shearing contribution is minimal and the transformation is mainly pure rotation. As any nominal beam location, 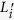, is a linear combination of 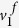 and 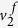, the actual beam location, *L*_*i*_, can be calculated from 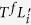.

Now that the basic mathematics to analyze the distortion have been outlined, the next step is to accurately measure 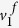 and 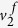 for all the STEM images. The lattice vector angles were measured using the projective standard deviation (PSD) method^23^. The basic idea of PSD is to project the STEM images for different orientations and calculate the standard deviation, *σ*, of the normalized projected line profile. If the image is projected along a lattice vector, the periodicity of the STEM image is preserved in the projected line profile, which leads to a large *σ*. Otherwise, the periodicity is averaged and the line profile leads to a very small *σ*. This works especially well for noisy STEM images and STEM images that only include several unit cells^14^. After determining the lattice vector angles, the lattice vector lengths are extracted from the projected line profile using a fast Fourier transform (FFT).

**Figure 4:**
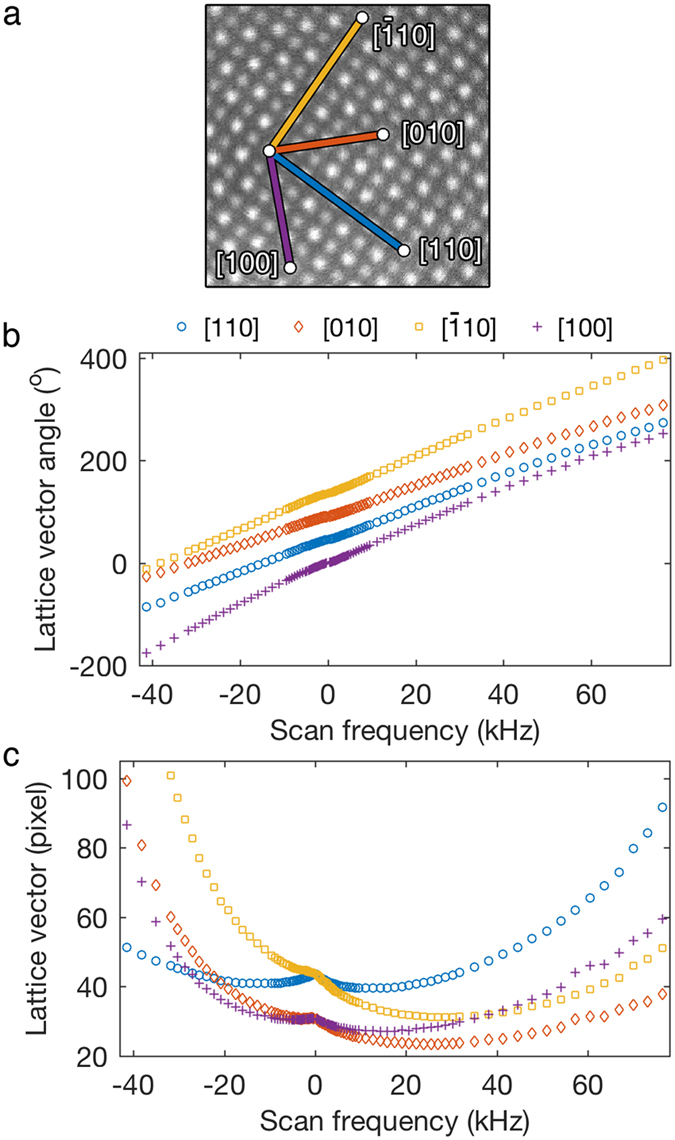
(**a**) Experimental STEM image acquired using Archimedean spiral with *f* = 318 Hz. The four low-index lattice vectors [100], [010], [110], and 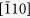 are overlaid on the image. The lattice vector angles (**b**) and lengths (**c**) are plotted as a function of frequency.

Figure 4b and c show the measured lattice vector angles and lattice vector lengths, respectively, for different frequencies, *f*. The four possible low index lattice vectors [100], [010], [110], and 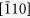 are indicated in Fig. 4a. All the lattice vector angles change monotonically as a function of *f* (Fig. 4b) and the lattice vector lengths behave more irregularly (Fig. 4c). For large |*f* |, the lattice vector lengths tend to increase and eventually, the lattice constants cannot be accurately measured from the STEM image (see Supplementary Fig. S2). Based on this behavior, only STEM images acquired using Archimedean spirals with frequencies between the largest negative frequency *f *^min^ = −41,380 Hz and positive frequency *f *^max^ = 76,394 Hz are considered.

**Figure 5:**
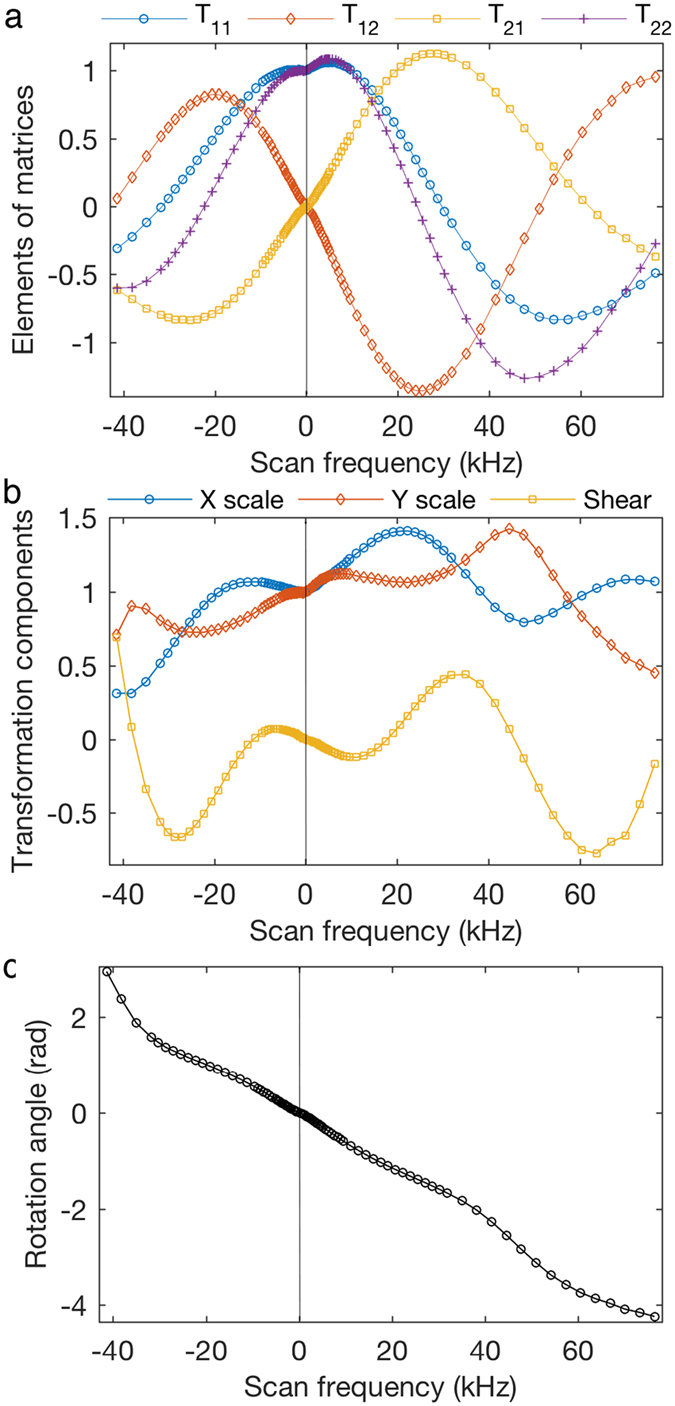
(**a**) The four elements of the 2D transformation matrices as a function of *f*. (**b**) X and Y scaling, and shearing components of the 2D linear transformation, as a function of *f*. (**c**) Rotation component of the 2D linear transformation as a function of *f*.

Using the two base vectors [110] and 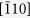 and equations (2) and (3), the transformation matrix, *T*^*f*^, was calculated for different *f*. The four elements in *T *^*f*^ are plotted as a function of *f* in Fig. 5a. These characteristic curves are the key parameters for beam control in spiral scans and all four curves appear periodic to some extent, which is mainly due to the rotation component of the *T*^* f*^. Further examination of the curves, however, reveals them to be quite complicated and an accurate numerical prediction requires extensive knowledge of the scan and lens systems for each individual microscope. The different behavior of spirals with positive *f* and negative *f* are also intriguing.

The rotation angle, *θ*^* f*^, shear component, *m *^*f*^, and scaling components, 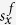 and 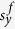, were calculated from the decomposition of *T*^* f*^,


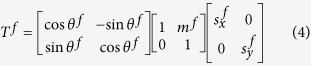


Figure 5b shows *m *^*f*^, 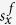, and 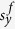 as a function of *f*, and Fig. 5c shows the *f*-dependent *θ*^* f*^. In the small *f* range (from −3 to ~3 kHz), 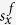 and 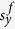 change slowly from 1, while *m*^*f*^ deviates slowly and linearly from 0. In this case the dominating transformation is *θ*^* f*^ that changes linearly with *f*, which is evident in Fig. 3b. *θ *^*f*^ continues to change monotonically with increasing *f*. On the other hand, *m *^*f*^, 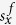, and 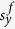 oscillate and change much faster for large *f*. For example, *m *^*f*^ varies more rapidly after the positive *f* hits a local minimum at 11 kHz and the negative *f* hits a local maximum at −7 kHz, resulting in significant shearing in the G-STEM images (see Fig. 3c, for example). When the beam moves too fast, the eventual combination of all the components causes too much distortion in the G-STEM image and renders them unsuitable for analysis.

### Beam control for spirals with varying frequency, *f*

A solution to the problem of non-uniform sampling density in Archimedean spirals is to use CLV spirals, which are defined by *X*(*t*) = *Y*(*t*) = *t*^0.5^ and *b *= 0.5 in equation (1)^20^. As discussed in ref. 15, a uniform sampling density is obtained when *X*(*t*) = *Y*(*t*) = *t*^0.5^. CLV spirals have a constant tangential velocity, but *ω* and *f* decrease from center to edge. Thus, the CLV spirals can be used to test the general applicability of the transformation matrices measured using the Archimedean spirals of constant *f*. Note that, unlike Archimedean spirals, *ω* in CLV spirals is simply a parameter and does not correspond directly to *f*. To avoid confusion, we use Ω to replace *ω* in Equation (1) for CLV spirals and use *f*_*i*_ for the position-dependent frequency. The radial distance between two adjacent scan rings is 2*π*/Ω, while the tangent distance along the tangential direction is Ω/2. Therefore, for isotropic sampling, the best Ω is 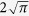 = 3.545.

**Figure 6:**
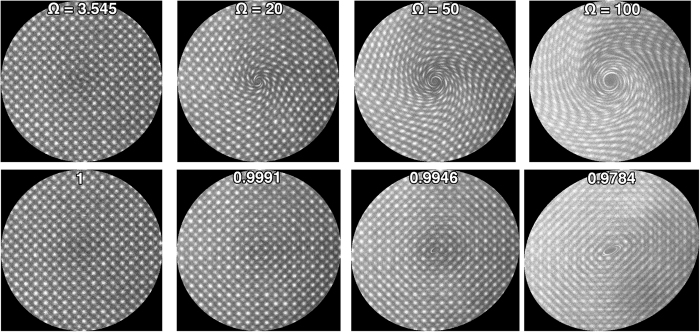
G-STEM images (top) acquired from [001] oriented STO using CLV spirals of different Ω. Distortion-corrected images (bottom) using transformation matrices determined from Archimedean spirals. Text annotations indicate the percentage of pixels with frequency in the range −41 kHz ~76 kHz.

Figure 6 (upper) shows reconstructed G-STEM images acquired using CLV spirals for different Ω. The frame time used was 1 s with a read-out frequency of 2 MHz. The reconstructed images are 512 × 512 pixels. The larger frequencies cause significant image distortions, turning the center of the scan into a ‘whirlpool’ for large Ω. The dark feature in the center is due to beam damage or contamination when the beam is not scanning, as the default scan system was set to (0, 0), for these experiments.

For image reconstruction, the frequency, *f*_*i*_, at each beam position, *L*_*i*_ = (*x*_*i*_, *y*_*i*_), was calculated as 
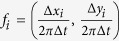
. The location-specific transformation matrix, 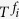, for each point was interpolated using the *T *^*f*^ measured from Archimedean spirals. When *f*_*i*_ > *f *^max^, the transformation matrix, 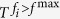, is approximated as 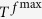. The lower part of Fig. 6 shows distortion-corrected G-STEM images as a function of *f*_*i*_. All images were restored to the correct geometry, demonstrating that the transformation matrices obtained from Archimedean spirals can be used to correct CLV spirals with varying frequencies. The image quality and SNR are uniform for CLV scans as a result of constant sampling density compared with Archimedean spirals. The streaking along <110> directions either crosses the whole image for large Ω (50 and 100) or is not present for small Ω (3.545 and 20). For each CLV spiral, *p*, the percentage of beam positions with frequency, *f*, falling in the range *f*_*i*_ ≤ *f *^max^, were calculated and are displayed on each distortion-corrected image in Fig. 6. A value of *p* = 1 indicates all the beam locations 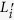 can be accurately corrected. When *p* is less than 1, the distortion correction in the center area is only approximate and residual distortions remain in the reconstructed image. For cases of Ω = 3.545 and 20, *p* is larger than 0.999 and there are no obvious residual distortions in the center of the image (Fig. 6). The elliptical residual distortion area in the center area enlarges as Ω increases and *p* decreases (image sequence left to right in bottom set of images in Fig. 6).

**Figure 7:**
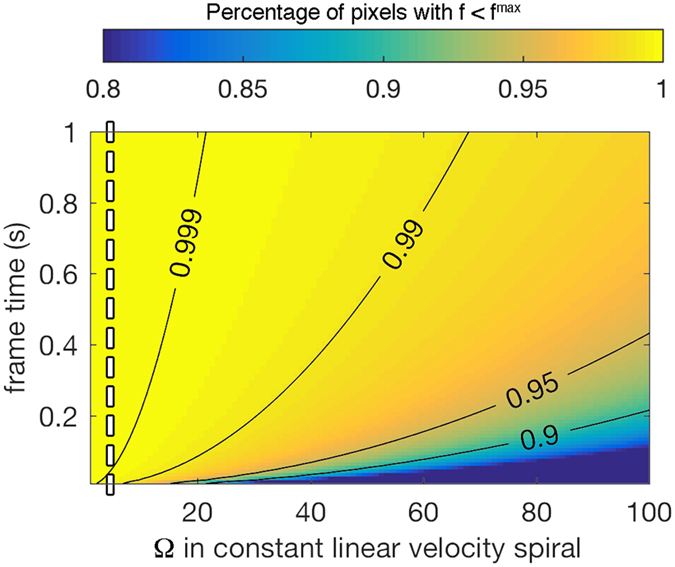
Percentage of pixels with frequency less than *f *^max^ for different Ω and frame time. The white dashed line corresponds to Ω = 3.545.

Careful selection of frame time and Ω will avoid a large residual distorted area in the center of the reconstructed image. Large frame times translate to the acquisition of more data points for the same scan area and imply a smaller frequency. Figure 7 shows the change in *p* as a function of frame time and Ω. The 0.999 contour line shown appears to be a good choice to eliminate residual distortions from a large frequency. When the optimum Ω = 3.545 is used, the frame time can be reduced to ~0.05 s without obvious distortion in the center of the image.

### Application to fast scans

One relevant and important application of the beam control G-STEM method is fast scanning, which is widely accepted as a primary means to reduce beam damage for beam sensitive materials^13^. In a conventional raster scan, the beam is deliberately settled at the beginning of each line to reduce the distortion associated with beam fly-back from the end of one line to the beginning of the next line. This settling time is called the fly-back time, which is typically on the order of 100 μs. The fly-back time wastes a significant portion of the frame time, especially for very fast frame acquisition, and can cause beam damage or contamination buildup along the left side of the image. Spiral scans, however, intrinsically avoid the fly-back problem since the beam movement is continuously smooth.

**Figure 8:**
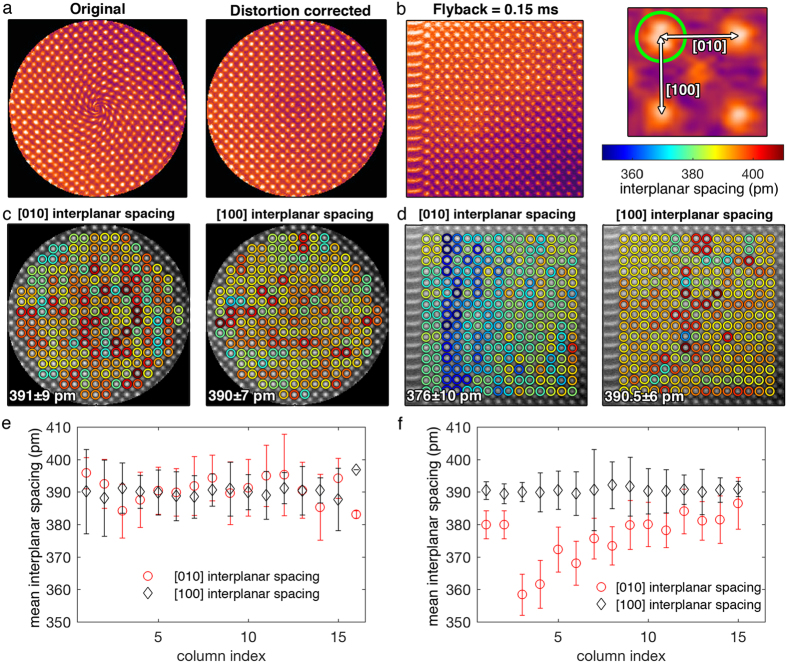
(**a**) Original reconstructed G-STEM image (left) and distortion-corrected G-STEM image (right) using a CLV spiral, frame time of 0.05 s and 30 frames. (**b**) G-STEM image using 200 × 200 raster scan with a 0.15 ms fly-back time. The right half shows measurement of [010] and [100] interplanar spacing from Sr NLN distances. (**c**,**d**) Unit-cell by unit-cell interplanar spacing maps ([100] (left) and [010] (right)) where the color of each circle is determined by the magnitude of the spacing for CLV spiral (**c**) and raster scan (**d**). Mean and standard deviation of interplanar spacing of columns of atoms from left to right for CLV spiral (**e**) and raster scan (**f**).

The performance of a CLV spiral scan and a raster scan are compared using a frame time of 0.05 s, 30 frames, and 2 MHz read-out frequency. The frame time can be further reduced if an external device with higher read-out frequency is used. For example, commercially available scans systems with readout frequencies of 5 MHz and significantly faster analog-to-digital converters are available. A 200 × 200 pixel image was reconstructed from the 100,000 data points acquired in each frame, and the final G-STEM image was averaged over 30 frames. The raster scan has 200 lines and 200 pixels (100 μs) per line and the fly-back time was 150 μs. The fly-back time was set up by parking the electron beam at the beginning of each line for 150 μs. Figure 8a shows the original, distortion-corrected G-STEM images. For isotropic sampling, Ω should always be set to 3.545. Based on Fig. 7, Ω = 3.545 also ensures that a fast frame time 0.05 s could be used without much distortion in the center (*p*~0.999). The distortion-corrected G-STEM image shows no obvious distortion in the center or streaking on the edge, i.e., the entire image exhibits the same quality. The raster scan image, however, is heavily influenced by the fly-back distortion, as evident by elongation of atoms on the left side of the image (Fig. 8b), despite the fact that fly-back constitutes 60% of the total frame time. Image distortion can be further visualized using Sr atom column nearest like-neighbor (NLN) distance maps^24^. The Sr atom columns were located using the method described in ref. 23. The NLN distances are directly measured to represent local [001] and [100] interplanar spacing. Results for both the CLV spiral and raster scan are presented as circles around atom columns using color codes that represent lengths of [001] and [100] spacing (Fig. 8c,d). Blue colors represent small spacing and red colors represent large spacing. The color distribution is uniform for the distortion-corrected CLV spiral image, whereas, the [010] map color changes from blue to yellow to red from left to right for the raster scan image, indicating that the [010] spacing increases from left to right.

To better understand this observed non-uniformity, the mean and standard deviations for NLN distances in each column of atoms are plotted as a function of the column index (Fig. 8e,f). The [010] and [100] interplanar spacings are nearly the same from left to right for the CLV spiral scan while the [010] lattice spacing increases from left to right for the raster scan. Moreover, the [010] interplanar spacing is much lower than [100] interplanar spacing, resulting from the actual beam location deviating from the nominal beam position after the sudden change during fly-back. This non-uniformity across the whole image from fly-back distortion makes any effort to correct them non-trivial, although some non-linear algorithms may restore the images at the price of additional processing time^12^,^16^. Therefore, although the image distortion from spiral scans seems to be more complicated than raster scanning, the GSTEM images could easily be corrected using the proposed method in this paper and are better for quantitative analysis as compared to raster scanning.

Practically, a single 0.05 s G-STEM frame already has sufficient quality for data interpretation (see Supplementary Fig. S3). Dynamics during *in situ* experiments can therefore be recorded using the G-STEM movies with at least 20 frames per second. Usage of higher read-out frequency (5 MHz instead of 2 MHz) would further push the recording speed to 50 frames per second. For a commercial system adapting the G-STEM method, the distortion corrected fast frames could be instantly generated using the characteristic curves pre-measured from Archimedean spirals. Similar to current software for raster scan, the users need to choose the frame time and the scan area to acquire G-STEM images with the scan path set to CLV and Ω to 3.545 for isotropic sampling.

In this paper, we discussed methods to accurately control a focused electron beam along spiral scanning pathways. The true beam location is related to the assigned beam location through 2D affine transformation matrices, which depend on the scan coil frequency. Archimedean spirals with constant frequency were used to experimentally measure the transformation matrices for different frequencies. We show that this approach can effectively remove beam scan distortions in spiral scans and the resultant G-STEM image quality is comparable to the traditional raster scan. For faster scans, CLV spirals outperform raster scans by reducing beam damage and image distortion from fly-back. We expect this approach will provide additional insights for precision electron beam control with high spatial and temporal resolution. Another promising application of beam control in STEM is electron beam induced modification of materials^25^,^26^, which has the potential to lead to the development of the next-generation of lithography or nanofabrication methods. For example, an atomic-scale, beam-induced amorphous to crystalline phase transformation in SrTiO_3_ was performed with 2 nm spatial resolution using an external beam control system^27^. Furthermore, precision control of electron beam irradiation can be used to induce and direct metal deposition from liquid phase precursors to form nanostructured architectures^26^,^28^.

## Methods

A [001] oriented SrTiO_3_ (STO) specimen was prepared using the focused ion beam (FIB) lift-out method. STEM images were acquired using an aberration-corrected Nion UltraSTEM 60–100, which is equipped with a cold field emission gun and operated at 60 kV. STEM images were acquired using a high angle annular dark field (HAADF) STEM detector and an electron probe convergence semi-angle of 31 mrad and a collection semi-angle range of 86–200 mrad. A custom field-programmable gate array (FPGA)-based system was developed to control the scan unit using LabView and customizable Matlab code. This system generates voltage waveforms that are sent to the x- and y- scan coils to enable controllable scanning pathways and dynamic beam positioning. The maximum readout frequency of the FPGA scan system is 2 MHz with an equivalent shortest collection interval of 0.5 μs. The G-STEM images are generated through a reconstruction process by assigning and averaging signals to a 2D grid based on the corresponding beam locations. More details of the system can be found in the literature^15^.

## Additional Information

**How to cite this article**: Sang, X. *et al*. Precision controlled atomic resolution scanning transmission electron microscopy using spiral scan pathways. *Sci. Rep.* **7**, 43585; doi: 10.1038/srep43585 (2017).

**Publisher's note:** Springer Nature remains neutral with regard to jurisdictional claims in published maps and institutional affiliations.

## Supplementary Material

Supplementary Information

## Figures and Tables

**Figure 1:**
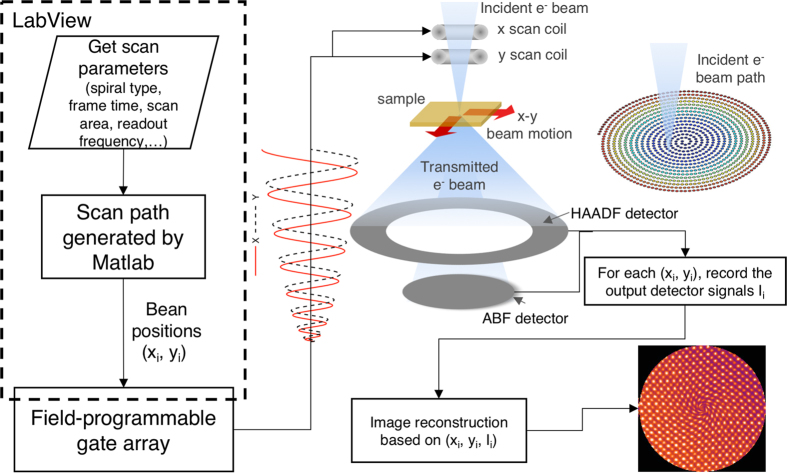
Schematic of the G-STEM method. A LabVIEW program (left) controls a field-programmable gate array to send beam position signals to the STEM scan coils (middle). The signals from HAADF and BF detectors are post-processed to form G-STEM images.
