# Corrigendum: Precision controlled atomic resolution scanning transmission electron microscopy using spiral scan pathways

**DOI:** 10.1038/srep45878

**Published:** 2017-04-10

**Authors:** Xiahan Sang, Andrew R. Lupini, Jilai Ding, Sergei V. Kalinin, Stephen Jesse, Raymond R. Unocic

Scientific Reports 7: Article number: 4358510.1038/srep43585; published online: 03 08 2017; updated: 04 10 2017

In this Article, an affiliation has been omitted for Jilai Ding. The correct affiliations are listed below:

Center for Nanophase Materials Science, Oak Ridge National Laboratory, Oak Ridge, TN 37831, USA.

School of Materials Science and Engineering, Georgia Institute of Technology, Atlanta, GA 30332, USA.

In addition, the Acknowledgements section in this Article is incomplete.

“Research supported by Oak Ridge National Laboratory’s (ORNL) Center for Nanophase Materials Sciences (CNMS), which is a U.S. Department of Energy (DOE), Office of Science User Facility (XS, JD, SVK, SJ, RRU), by the Division of Materials Sciences and Engineering, Office of Basic Energy Sciences, DOE (ARL) and by ORNL’s Laboratory Directed Research and Development Program, which is managed by UT-Battelle LLC for the U.S. DOE (SJ)”.

should read:

“Research supported by Oak Ridge National Laboratory’s (ORNL) Center for Nanophase Materials Sciences (CNMS), which is a U.S. Department of Energy (DOE), Office of Science User Facility (XS, SVK, RRU), by the Division of Materials Sciences and Engineering, Office of Basic Energy Sciences, DOE (ARL), by the ORNL GO! Fellowship program (JD) and by ORNL’s Laboratory Directed Research and Development Program, which is managed by UT-Battelle LLC for the U.S. DOE (SJ)”.

