# Supplementary material for: Precision controlled atomic resolution scanning transmission electron microscopy using spiral scan pathways
Source: Sci Rep. 2017 Mar 8;7:43585. doi: 10.1038/srep43585 (PMC5341089; doi:10.1038/srep43585)
Supplement: Supplementary Information [file srep43585-s1.pdf]

Precision controlled atomic resolution scanning transmission electron microscopy using spiral scan pathways

Xiahan Sang[1,3], Andrew R Lupini[2,3], Jilai Ding[1], Sergei V. Kalinin[1,3], Stephen Jesse[1,3], Raymond R. Unocic[1,3]

[1]Center for Nanophase Materials Sciences
Oak Ridge National Laboratory, Oak Ridge, TN, 37831 USA

[2]Materials Science and Technology Division
Oak Ridge National Laboratory, Oak Ridge, TN, 37831 USA

[3]Institute for Functional Imaging of Materials
Oak Ridge National Laboratory, Oak Ridge, TN 37831 USA

Corresponding Authors:
Email: sangx@ornl.gov

Displacement between the four quadrants in square spiral scan using different scan frequency

The scan distortion for STEM images acquired using square spiral scans depends on the scan frequency. In Fig. S1 we show images acquired with different square spirals with ten times different frame time, therefore different scan frequency. Images acquired with 0.2 s frame time shows significant scan distortion especially between the four quadrants. Therefore, the scan distortion depends on the scan frequency.

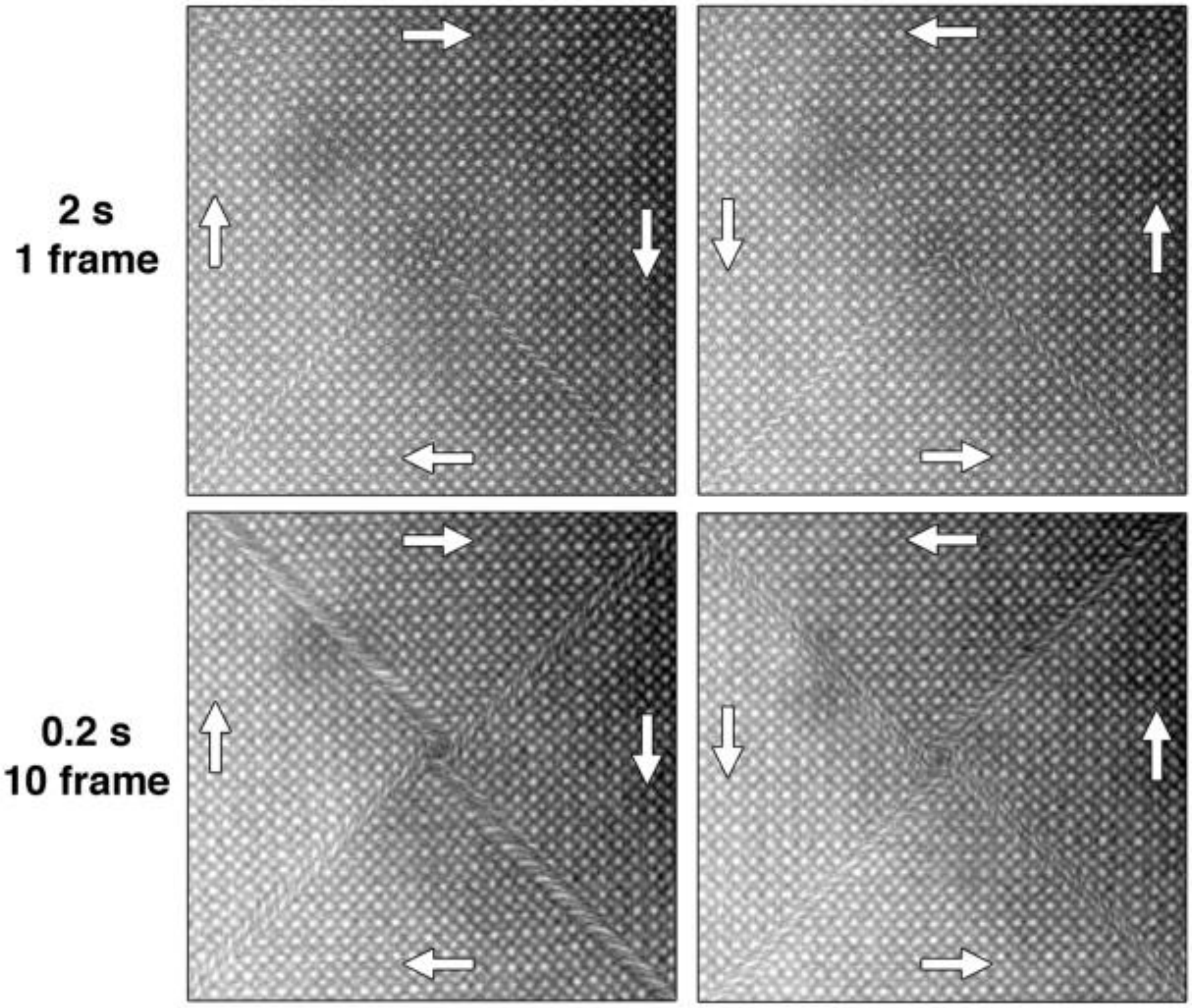

Figure S1. G-STEM images acquired from STO [001] using two square spiral scans of two different conditions: (a) a single frame with 2 s frame time; (b) average of ten fast frames with 0.2 s frame time.

G-STEM images acquired using very large scan frequency

In Fig. S2 we show reconstructed G-STEM images acquired using Archimedean spirals with very large negative and positive scan frequency f. When f < -41 kHz or f > 76 kHz, the reconstructed images are not suitable for image distortion analysis.

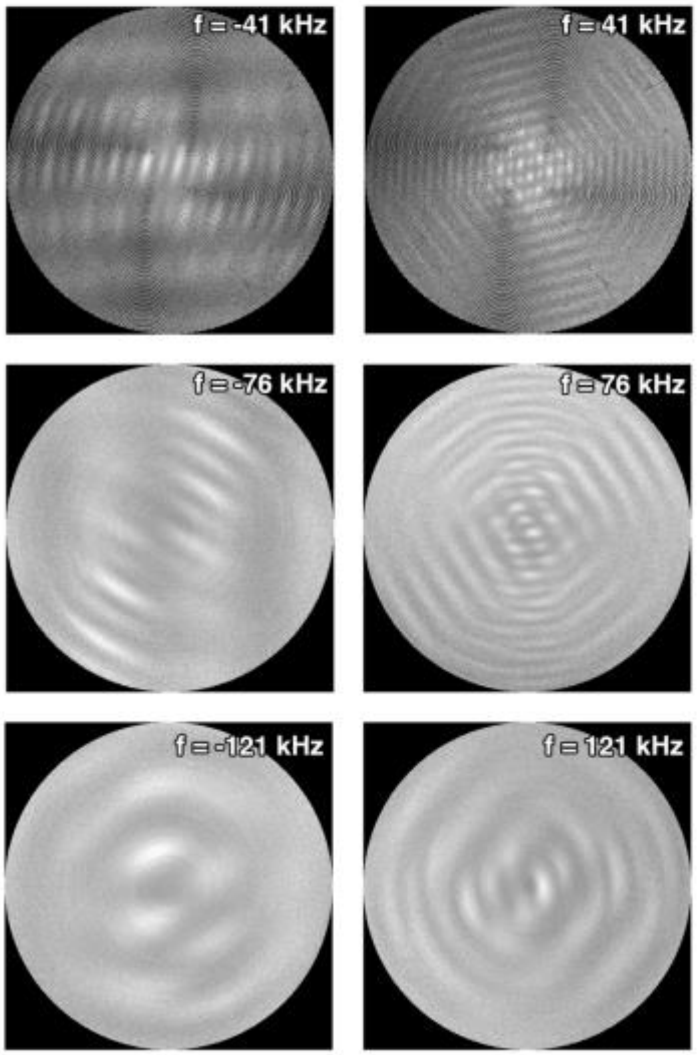

Figure S2: Reconstructed GSTEM images acquired along STO [001] using Archimedean spirals of very large scan frequencies.

A single frame G-STEM image

For better signal to noise ratio, the images shown in Fig. 8 were averaged from 30 frames. However, a single frame image also contains atomic information after image processing. Fig. S3 shows the first frame of the CLV dataset after blurred using a Gaussian distribution with a standard deviation of 1 pixel. The bright Sr atoms and the dark Ti atoms are clearly visible from this single frame.

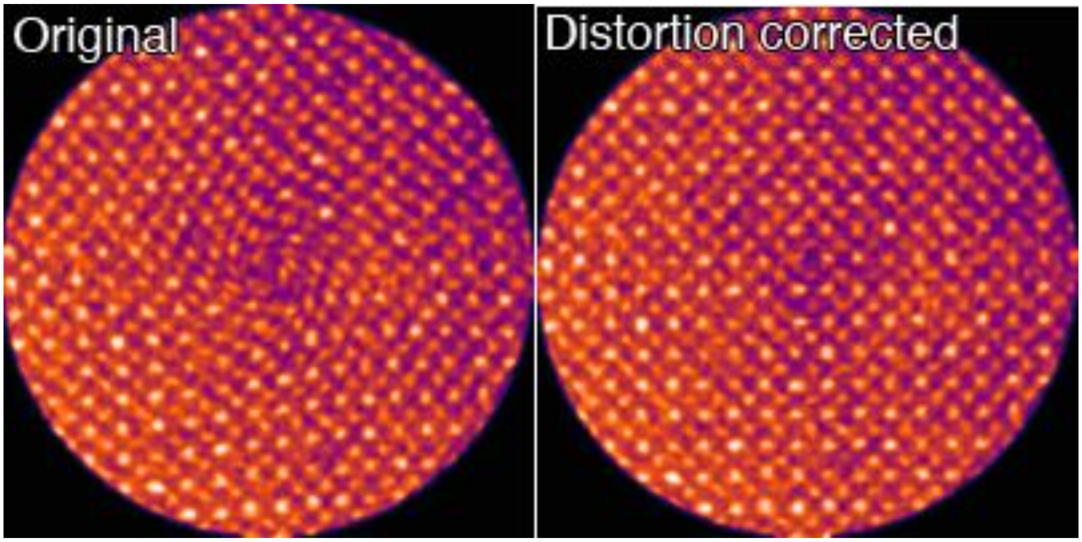

Figure S3. The original and distortion-corrected G-STEM image acquired using CLV spiral with a frame time of 0.05. This image is the first frame of the dataset with 30 frames used in Fig. 8.
